# Generalizability of the Progressive Resistance Training versus Total Hip Arthroplasty (PROHIP) trial: a cross-sectional study of 402 patients in Denmark

**DOI:** 10.2340/17453674.2025.44756

**Published:** 2025-09-17

**Authors:** Thomas FRYDENDAL, Robin CHRISTENSEN, Inger MECHLENBURG, Lone Ramer MIKKELSEN, Claus VARNUM, Manuel Josef BIEDER, Stig Storgaard JAKOBSEN, Søren Overgaard, Kim Gordon INGWERSEN

**Affiliations:** 1 Department of Physiotherapy, Lillebaelt Hospital – University Hospital of Southern Denmark, Vejle Hospital, Vejle; 2 Department of Clinical Research, University of Southern Denmark, Odense; 3 Section for Biostatistics and Evidence-Based Research, the Parker Institute, Bispebjerg and Frederiksberg Hospital; 4 Research Unit of Rheumatology, Department of Clinical Research, University of Southern Denmark, Odense University Hospital, Odense; 5 Department of Orthopedic Surgery, Aarhus University Hospital, Aarhus; 6 Department of Clinical Medicine, Aarhus University, Aarhus; 7 Elective Surgery Centre, Silkeborg Regional Hospital, Silkeborg; 8 Department of Orthopedic Surgery, Lillebaelt Hospital – University Hospital of Southern Denmark, Vejle Hospital, Vejle; 9 Department of Regional Health Research, University of Southern Denmark, Odense; 10 Department of Orthopedic Surgery, Næstved Hospital, Næstved; 11 Department of Orthopedic Surgery and Traumatology, Odense University Hospital, Odense; 12 Department of Orthopedic Surgery and Traumatology, Copenhagen University Hospital, Bispebjerg Hospital, Copenhagen; 13 Department of Clinical Medicine, Faculty of Health and Medical Sciences, University of Copenhagen, Copenhagen, Denmark

## Abstract

**Background and purpose:**

There is ongoing debate over whether results from randomized trials assigning patients to surgery or first-line treatment can be generalized to clinical practice. Therefore, we aimed to compare patients with hip osteoarthritis accepting enrollment in the Progressive Resistance Training versus Total Hip Arthroplasty (PROHIP) trial in Denmark with those declining (enrolled in an observational cohort [non-PROHIP]).

**Methods:**

We used a cross-sectional study design to compare demographics and patient-reported outcomes among patients eligible for enrollment in the PROHIP trial. We used the standardized difference (StdDiff), the absolute difference with 95% confidence interval (CI), and the propensity (odds ratio [OR]) of accepting participation in the PROHIP trial to assess imbalances between groups. We pre-specified that StdDiff values < 0.2 indicated a negligible difference, whereas values ≥ 0.8 indicated incomparability.

**Results:**

402 patients were included, with 109 in the PROHIP trial and 293 in the non-PROHIP cohort. Patients enrolled in the PROHIP trial had a mean (standard deviation [SD]) Oxford Hip Score at baseline of 25.1 (SD 5.9) compared with 22.6 (SD 6.9) in the non-PROHIP cohort (between-group difference, 2.5 points [CI 1.1–4.0], StdDiff 0.4, OR 1.06 [CI 1.02–1.10]). This pattern was consistent across almost all secondary patient-reported outcomes applied in the PROHIP trial. For most demographic variables, there were negligible between-group differences at baseline.

**Conclusion:**

We found minimal imbalances in some baseline demographic variables and most patient-reported outcomes, with those who accepted enrollment in the PROHIP trial having more favorable outcomes at recruitment than those who declined. However, most differences were not clinically important.

Hip osteoarthritis is a major contributor to disability and a leading cause for total hip arthroplasty (THA) [1,2]. This surgical procedure is considered an effective treatment for reducing hip pain and functional impairments and improving quality of life in patients with severe hip osteoarthritis [1]. Although more than 1 million THAs are performed annually worldwide [1], randomized controlled trials comparing this procedure’s effectiveness with first-line treatment have been lacking [3]. Across clinical guidelines, exercise such as resistance training is consistently recommended as a first-line treatment for hip osteoarthritis [4].

We recently reported results from the Progressive Resistance Training versus Total Hip Arthroplasty randomized trial (PROHIP), which showed that THA resulted in clinically important larger reductions in patient-reported hip pain and improvements in function at 6 months as compared with resistance training [5]. However, our trial had a low enrollment rate with only 7% of all patients who were assessed for eligibility accepting participation [5]. Enrollment rates in previous randomized controlled trials comparing surgical procedures with first-line treatment have varied between 7% and 22% [6-8]. This issue may potentially limit the generalizability of the results from randomized controlled trials to clinical practice [9,10].

To address this, we established a parallel observational cohort consisting of eligible patients who declined participation in the PROHIP trial (non-PROHIP) [11]. We aimed to compare baseline demographics and patient-reported outcome scores between patients who enrolled in the PROHIP trial with those in the non-PROHIP observational cohort to exploratorily investigate generalizability.

## Methods

### Study design

We used a cross-sectional study design to collect and compare demographics and patient-reported outcome scores from patients with severe hip osteoarthritis and eligible for enrollment in the PROHIP trial at baseline [11]. This study is reported in agreement with the Strengthening the Reporting of Observational Studies in Epidemiology (STROBE) statement for cross-sectional studies, and the statistical analysis plan (see Supplementary data) was made publicly available at ClinicalTrials.gov (August 16, 2022: NCT04070027) before any analyses commenced.

### Setting

Patients were enrolled from the orthopedic departments at Vejle Hospital and Odense University Hospital in the Region of Southern Denmark, Aarhus University Hospital in the Central Denmark Region, and Næstved Hospital in Region Zealand from September 2, 2019 until the predefined recruitment deadline for patient enrollment was reached on June 30, 2021 [11].

### Patients

Orthopedic surgeons prospectively screened patients for eligibility during medical consultations in accordance with standard hospital-specific clinical procedures. Patients 50 years of age or above with severe hip osteoarthritis and indication for THA based on hip pain, clinical presentation, and radiographic imaging (joint space width below 2 mm) were considered eligible for enrollment. Exclusion criteria were severe walking deficits (dependency on 2 crutches or walker); a body mass index >35; fracture of the leg or foot within previous 12 months; planned surgery on the leg or foot in the following 6 months; cancer diagnosis and current receipt of chemo-, immuno- or radiotherapy; neurological disease; and other reasons such as inability to understand Danish or being considered mentally or physically unable to participate. Eligible patients were briefly informed about the PROHIP trial by the orthopedic surgeon and given the option of receiving detailed standardized information provided by research coordinators. Eligible patients who accepted enrollment in the PROHIP trial completed baseline measurements and were afterwards randomized to receive either a THA or a resistance training program. Eligible patients who declined to receive detailed information or participation were invited into a parallel prospective non-interventional observational cohort, and those who accepted enrollment completed baseline measurements and were subsequently scheduled for THA. The enrollment procedures are described in full detail in the trial protocol [11].

### Baseline demographics

Demographic variables included sex, age, height, weight, body mass index, educational level beyond high school, employment, current smoker, alcohol intake above 10 units per week, affected hip on right side, duration of hip symptoms, previous THA, previous total knee arthroplasty (TKA), previous treatment due to hip symptoms, use of analgesics due to hip pain, and comorbidities.

### Patient-reported outcome scores

As also specified in the PROHIP trial [5,11], the primary outcome variable was patient-reported hip pain and function measured using the Oxford Hip Score (OHS) [12]. This 12-item patient-reported questionnaire provides a total score ranging from 0 to 48, with higher scores indicating less pain and better function, and the minimal important difference (MID) is estimated to be 5 points [13].

Secondary outcome variables included patient-reported domains of hip pain, hip symptoms, function in activities of daily living, hip-related quality of life, and function in sports and recreation measured using the Hip disability and Osteoarthritis Outcome Score (HOOS) subscales (range, 0 [worst] to 100 [best], MID = 10 points) [14]. Another secondary outcome score variable was patient-reported physical activity level measured using the single-item University of California Los Angeles (UCLA) activity score (range, 1 [inactive] to 10 [regular participation in impact sport or heavy labor], MID = 2 points) [15].

Additional outcome variables were patient-reported hip pain intensity at rest and during activities measured using the single-item Visual Analogue Scale (VAS) score (score, 0 [no pain] to 100 [worst pain imaginable], MID = 15.3 points) [16], health-related quality of life as assessed by the EuroQol Group 5-dimension (EQ-5D-5L) index score (range, –0.76 [worst] to 1.00 [best] using a Danish value set, MID = 0.4 points), and overall health status as assessed by the EuroQol Group Visual Analogue Scale (EQ-VAS) score (range, 0 [worst imaginable health] to 100 [best imaginable health], MID = 9.3 points [17].

### Data collection procedures

Baseline demographic and patient-reported outcome variables were collected using online questionnaires in an electronic management system. Patients enrolled in the PROHIP trial completed the questionnaires in an undisturbed examination room at the hospitals with the possibility of asking clarifying questions, while anthropometric measures of height and weight were conducted by an outcome assessor. Patients accepting participation in the non-PROHIP cohort received an email containing a link to the online questionnaires and completed them at home for pragmatic reasons.

### Sample size considerations

As the enrollment ratio in PROHIP and non-PROHIP was unknown, we did not conduct a formal sample size and power calculation for this cross-sectional study. Based on a previous randomized controlled trial comparing TKA with first-line treatment [8], we anticipated an enrollment rate of about 7% of all patients assessed for eligibility in PROHIP. This yielded that 1,720 patients were probably needed for eligibility assessment to enroll the sample size of 120 patients in PROHIP, as estimated previously in the trial protocol [11]. We expected a 2 (14% ≈ 240 patients) to 4 (28% ≈ 480 patients) times higher enrollment rate in non-PROHIP, resulting in an approximated total sample size between 360 and 600 patients for this cross-sectional study.

### Statistics

Based on the intention-to-infer from population [18], all baseline demographics and patient-reported outcomes for each group are presented with means and standard deviations (SD) for continuous variables, and numbers and proportions (%) for categorical variables. The assumption of normality of the continuous variables was assessed using visual inspection of quantile–quantile plots and histograms.

The statistical approach to evaluate generalizability is not immediately clear from the literature. Guidance is available, however, providing statistical measures that could be used to compare results from an exposed group (i.e., PROHIP) to a second sample—referred to as the broader target population (i.e., non-PROHIP) [19]. To exclude the possibility of selective outcome reporting, the preferred methods should involve comparing all characteristics collected in the randomized controlled trial with those of the target population. To evaluate whether significant differences exist between the PROHIP and non-PROHIP groups that could reduce generalizability, we applied 3 different measures that are feasible for 2-group comparisons: the standardized difference (StdDiff), the absolute difference with confidence interval (in the actual units), and the propensity (i.e., odds) of accepting participation in the PROHIP trial [18]. As suggested previously [19], we evaluated the comparability of the PROHIP trial and non-PROHIP groups using balance diagnostics to describe possible differences between groups. In this approach, we compared means and proportions of baseline demographics and patient-reported outcomes using StdDiff (see Supplementary data). We interpreted a StdDiff of < 0.2 as indicating a negligible difference in the baseline variable between the 2 groups, while a StdDiff of 0.8 or greater was considered definitive incomparability (i.e., indicative of low generalizability). Between-group differences are presented with means and 95% confidence intervals (CI). As pre-specified in our trial protocol [11], for the primary outcome measure in the PROHIP trial the OHS, we interpreted a 95% confidence interval excluding a difference greater than 5 points between groups as indicating absence of a clinically important difference. As our aim was to conduct an exploratory investigation of generalizability and because we lacked a theoretical basis for testing a series of null hypotheses, we did not find it necessary to adjust for multiple comparisons in this instance. Therefore, all CIs presented are applicable only for interpreting whether the MID is included; they were not adjusted for multiplicity and should not be used in place of hypothesis testing. Finally, we used univariable logistic regression to calculate the propensity of accepting enrollment in the PROHIP trial compared with those who declined by estimating the odds ratio (OR).

All statistical analyses and estimations were performed using STATA 17.0 (StataCorp, College Station, TX, USA).

### Ethics, registration, data sharing plan, funding, and disclosures

The PROHIP trial was approved by the Regional Committees on Health Research Ethics for Southern Denmark (Project-ID: S-20180158) and the Danish Data Protection Agency (Journal No 19/20337), and registered at ClinicalTrials.gov (NCT04070027). Written informed consent was obtained from each patient in accordance with the Helsinki Declaration. Data is available upon reasonable request to the author group. This study was funded by the Danish Rheumatism Association (R153-A4771, R161-A5280, R166-A5553, R176-A6231), Region of Southern Denmark (17/33622, 18/41994), Region Zealand and Region of Southern Denmark (R23-A786), the Association of Danish Physiotherapists Research Fund (N/A), The Research Council at Næstved–Slagelse–Ringsted Hospitals (N/A), and the A.P. Moeller Foundation (N/A). The Section for Biostatistics and Evidence-Based Research the Parker Institute, Bispebjerg and Frederiksberg Hospital is supported by a core grant from the Oak Foundation (OCAY-18-774-OFIL). The funders were not involved in designing the study, data collection, data management, data analysis, interpretation of the results, writing the article, or in the decision to submit the article for publication. TF, RC, IM, LRM, SSJ, MJB, and KGI declare no conflicts of interest. CV received travel expenses from Stryker with no relevance to this study. SO received payment for lectures from Johnson & Johnson and Heraeus with no relevance to this study. Complete disclosure of interest forms according to ICMJE are available on the article page, doi: 10.2340/17453674.2025.44756

## Results

### Patient enrollment

**Figure 1:**
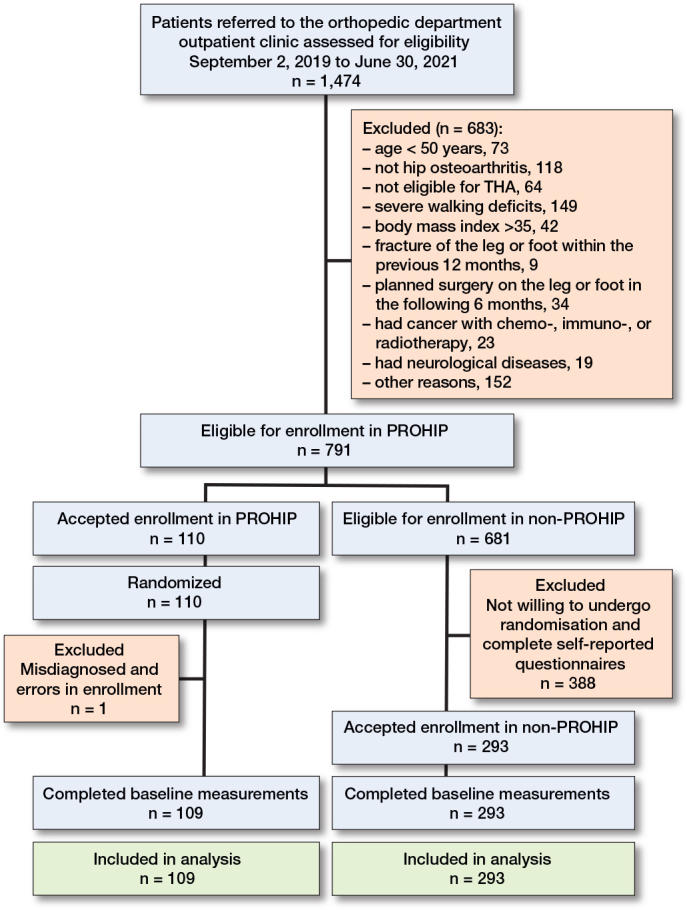
Flowchart of the patients in the study. Progressive Resistance Training versus Total Hip Arthroplasty (PROHIP) trial. Parallel prospective non-interventional observational cohort (non-PROHIP).

**Figure 2:**
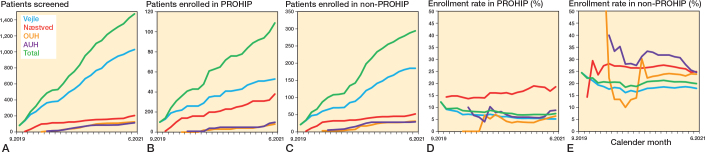
(A) Number of patients assessed for eligibility; (B) number of patients enrolled in the Progressive Resistance Training versus Total Hip Arthroplasty (PROHIP) trial; (C) number of patients enrolled in the parallel prospective non-interventional observational cohort (non-PROHIP); (D) enrollment rate in PROHIP; and (E) enrollment rate in non-PROHIP.

We assessed 1,474 patients for eligibility and 791 (54%) were considered eligible for enrollment. Of these patients, 109 (14%) were enrolled in the PROHIP trial. For the remaining 681 patients, 293 (43%) were enrolled in the non-PROHIP cohort. As a result, 402 patients completed baseline measurements and were included in the main analysis (Figure 1). The enrollment of all patients assessed for eligibility was 7% in the PROHIP trial and 20% in the non-PROHIP cohort, corresponding to an enrollment ratio of 1:2.7 (Figure 2).

### Demographic variables

For the majority of demographic variables, there were negligible imbalances differences between the 2 groups at baseline, but patients who accepted enrollment in the PROHIP trial had a higher body mass index, and fewer had previously received a joint arthroplasty (i.e., THA on the opposite side and/or TKA on either side), supervised exercise, and/or an glucocorticoid injection than those in the non-PROHIP cohort (StdDiff 0.2–0.4) (Table 1).

**Table 1:** Demographic variables at baseline in patients enrolled in the Progressive Resistance Training versus Total Hip Arthroplasty (PROHIP) trial and a parallel prospective non-interventional observational cohort (non-PROHIP). Values are count (%) or mean (SD) as specified

Demographic variables	Baseline		Between-group difference		
	PROHIP	non-PROHIP	Mean	StdDiff	Odds ratio ^a^ (CI)
	(n = 109)	(n = 293)	difference (CI)		
Female sex	54 (50)	147 (50)	0 (–10 to 11)	0.0	0.98 (0.63–1.51)
Age (SD)	67.6 (7.2)	68.2 (7.9)	0.6 (–1.1 to 2.3)	0.1	0.99 (0.96–1.02)
Height, m (SD) **^b^**	1.7 (0.1)	1.7 (0.1)	0.0 (0.0 to 0.0)	0.2	0.97 (0.95–1.00)
Weight, kg (SD) **^b^**	81.9 (14.2)	79.8 (15.1)	2.1 (–1.2 to 5.4)	0.1	1.01 (0.99–1.02)
Body mass index (SD) **^b^**	28.1 (3.8)	26.7 (4.0)	1.4 (0.5 to 2.3)	0.4	1.09 (1.03–1.15)
Education level above high school	57 (52)	163 (55)	3 (–8 to 14)	0.1	0.87 (0.56–1.36)
Employment					
Employed for wages or self-employed	35 (32)	96 (33)	1 (–10 to 11)	0.0	0.97 (0.61–1.55)
On sick leave	3 (2.8)	5 (1.7)	1 (–2 to 4)	0.1	1.63 (0.38–6.94)
Retired	70 (64)	179 (61)	3 (–8 to 14)	0.1	1.14 (0.72–1.80)
Other status	1 (0.9)	13 (4.4)	3 (0 to 6)	0.2	0.20 (0.03–1.54)
Current smoking	5 (4.6)	30 (10)	6 (0 to 11)	0.2	0.42 (0.16–1.12)
Alcohol intake >10 units per week ^**c**^	13 (12)	22 (7.5)	4 (–2 to 11)	0.1	1.66 (0.81–3.44)
Affected hip on right side	62 (57)	150 (51)	6 (–5 to 17)	0.1	1.26 (0.81–1.96)
Duration of hip symptoms, years (SD)	2.3 (2.1)	2.3 (2.8)	0.0 (–0.6 to 0.6)	0.0	1.00 (0.92–1.09)
Previous total hip arthroplasty	11 (10)	69 (23)	13 (6 to 21)	0.4	0.36 (0.18–0.72)
Previous total knee arthroplasty	2 (1.8)	24 (8)	6 (–2 to 10)	0.3	0.21 (0.05–0.90)
Previous treatment for hip-related pain					
Supervised exercise	31 (28)	115 (39)	11 (1 to 21)	0.2	0.62 (0.39–0.99)
Manual and/or passive treatment	17 (15)	64 (21)	6 (–2 to 14)	0.2	0.66 (0.37–1.19)
Corticosteroid injection	3 (2.8)	22 (8.2)	5 (1 to 9)	0.2	0.35 (0.10–1.19)
Other non-surgical treatment	13 (12)	58 (20)	8 (0 to 16)	0.2	0.55 (0.29–1.05)
Use of analgesics for hip-related pain					
Paracetamol	83 (76)	234 (80)	4 (–5 to 13)	0.1	0.80 (0.48–1.36)
Nonsteroidal anti-inflammatory drug	39 (36)	88 (30)	6 (–5 to 16)	0.1	1.29 (0.82–2.07)
Opioids	8 (7.3)	21 (7.2)	0 (–6 to 6)	0.0	1.03 (0.44–2.29)
Other analgesic agent	9 (8.3)	19 (6.5)	2 (–4 to 8)	0.1	1.30 (0.57–2.96)
Comorbidities					
0	55 (50)	144 (49)	1 (–10 to 12)	0.0	1.05 (0.68–1.64)
1	20 (18)	62 (21)	3 (–6 to 12)	0.1	0.84 (0.48–1.47)
2	17 (16)	52 (18)	2 (–6 to 10)	0.1	0.86 (0.47–1.56)
≥3	17 (16)	35 (12)	4 (–4 to 12)	0.1	1.36 (0.73–2.55)

I = 95% confidence intervals; SD = standard deviation.

StdDiff = standardized difference with a value of less than 0.2 indicates a negligible difference in the baseline variable between the 2 groups, while a value of 0.8 or greater indicates definitive incomparability (i.e., indicative of low generalizability).

a Odds ratio with CI on all univariable logistic regression models reflecting the impact on a propensity score.

b In non-PROHIP, 4 patients had missing data on height, weight, and body mass index.

c One unit equals 10 mL or 8 g of pure alcohol.

### Patient-reported outcome variables

For the primary outcome measure, patients who accepted enrollment in the PROHIP trial had a mean OHS at baseline of 25.1 (SD 5.9) compared with 22.6 (SD 6.9) in patients in the non-PROHIP cohort (between-group difference 2.5 points, CI 1.1–4.0), corresponding to a StdDiff of 0.4. This indicates the absence of a clinically important difference, as the CI excluded differences greater than 5 points between the 2 groups.

**Figure 3:**
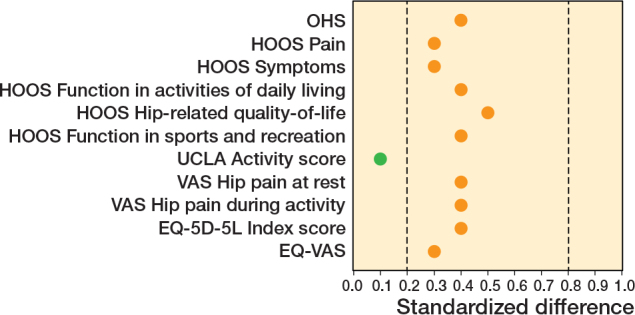
Standardized differences of the means across the different patient-reported outcomes score variables between patients in the Progressive Resistance Training versus Total Hip Arthroplasty (PROHIP) trial and parallel prospective non-interventional observational cohort (non-PROHIP) at baseline. Standardized differences of less than 0.2 indicate a negligible difference, while 0.8 or greater indicates definitive incomparability (i.e., indicative of low generalizability) in the patient-reported outcome score variables between the 2 groups. OHS: Oxford Hip Score; HOOS: Hip disability and Osteoarthritis Outcome Score; UCLA: University of California Los Angeles (UCLA) activity score; VAS: Visual Analogue Scale; EQ-5D-5L: EuroQol Group 5-dimensions 5-levels; and EQ-VAS: the EuroQol Group Visual Analogue Scale.

For all the secondary outcome measure domains, except for the UCLA activity score (StdDiff 0.1), a consistent pattern was noted, displaying imbalances between the 2 groups with more favorable scores indicating less severe symptoms in patients accepting enrollment in the PROHIP trial compared with those declining in the non-PROHIP cohort (StdDiff 0.3–0.5). As the CIs for the HOOS function in activities of daily living, hip-related quality of life, and function in sports and recreation subscales, as well as the EQ-VAS, included between-group differences greater than the MIDs, these were interpreted as indicating a possible clinically important difference (Table 2, Figure 3).

**Table 2:** . Patient-reported outcome scores at baseline in patients enrolled in the Progressive Resistance Training versus Total Hip Arthroplasty (PROHIP) trial and parallel prospective non-interventional observational cohort (non-PROHIP). Values are Mean (SD)

Patient-reported outcome variables	Baseline scores, mean (SD)		Between-group difference		
	PROHIP	non-PROHIP	Mean	StdDiff	Odds ratio ^a^ (CI)
	(n = 109)	(n = 293)	difference (CI)		
Primary outcome					
OHS **^a^**	25.1 (5.9)	22.6 (6.9)	2.5 (1.1 to 4.0)	0.4	1.06 (1.02–1.10)
Secondary outcomes					
HOOS subscales **^b^**					
Pain	47.8 (15.0)	42.9 (15.9)	4.9 (1.4 to 8.3)	0.3	1.02 (1.01–1.03)
Symptoms	45.8 (17.7)	40.2 (16.4)	5.6 (1.9 to 9.3)	0.3	1.02 (1.01–1.03)
Function in activities of daily living	54.1 (17.4)	46.7 (17.9)	7.4 (3.5 to 11.3)	0.4	1.02 (1.01–1.04)
Hip-related quality of life	34.0 (15.4)	26.3 (14.7)	7.7 (4.4 to 11.0)	0.5	1.03 (1.02–1.05)
Function in sports and recreation	31.5 (19.8)	23.4 (18.1)	8.1 (4.0 to 12.2)	0.4	1.02 (1.01–1.03)
UCLA activity score **^c^**	5.2 (1.7)	5.0 (2.0)	0.2 (–0.2 to 0.6)	0.1	1.06 (0.95–1.19)
VAS hip pain at rest **^d^**	41.5 (22.5)	50.6 (23.6)	9.1 (4.0 to 14.2)	0.4	0.98 (0.97–0.99)
VAS hip pain during activity **^d^**	64.8 (16.4)	71.1 (16.5)	6.3 (2.7 to 10.0)	0.4	0.98 (0.97–0.99)
EQ-5D-5L index score **^e^**	0.67 (0.23)	0.57 (0.27)	0.10 (0.04 to 0.16)	0.4	1.02 (1.01–1.03)
EQ-VAS **^f^**	60.4 (21.5)	52.9 (22.7)	7.5 (2.6 to 12.5)	0.3	1.02 (1.01–1.03)

For abbreviations, see Table 1.

a The Oxford Hip Score (OHS) ranges from 0 (worst) to 48 (best).

b The Hip disability and Osteoarthritis Outcome Score (HOOS) subscales range from 0 (worst) to 100 (best).

c The University of California Los Angeles (UCLA) activity score ranges from 1 (inactive) to 10 (regular participation in impact sport or heavy labor).

d The Visual Analogue Scale (VAS) ranges from 0 (no pain) to 100 (worst pain imaginable).

e The EuroQol Group 5-dimensions 5-levels (EQ-5D-5L) index score ranges from –0.76 (worst) to 1.00 (best).

f The EuroQol Group Visual Analogue Scale (EQ-VAS) ranges from 0 (worst) to 100 (best).

### Propensity of accepting enrollment in the PROHIP trial

Among eligible patients, those who were more likely to accept enrollment in the PROHIP trial were those with a higher body mass index, no previous receipt of THA, TKA, and/or supervised exercise, and more favorable scores on almost all the patient-reported outcome measures. Based on the propensity to accept enrollment in the PROHIP trial, patients with higher OHS at baseline, indicating less hip pain and better function, were more inclined to accept enrollment (OR 1.06, CI 1.02–1.10).

## Discussion

We aimed to compare baseline demographics and patient-reported outcome scores between patients who enrolled in the PROHIP trial with those in the non-PROHIP observational cohort to exploratorily investigate generalizability. We found that patients 50 years of age or older with severe hip osteoarthritis and an indication for surgery who accepted enrollment in the PROHIP trial had slightly different demographics with a higher body mass index and less experience with previous joint arthroplasty surgery and supervised exercise, as well as slightly more favorable patient-reported outcomes indicating less severe symptoms for almost all domains at baseline than those who declined participation in the PROHIP trial. However, the between-group differences were only considered possibly clinically important for the individual domains of function in activities of daily living, hip-related quality-of-life, function in sports and recreation, and overall health status. Therefore, although imbalances were found between the 2 groups, our findings indicate that the patients enrolled in the PROHIP trial appear to be largely representative of patients undergoing THA in clinical practice. While these findings are specific to the PROHIP trial, they may offer insights into patient enrollment and representativeness that could be relevant to other randomized trials in orthopedic surgery, especially those including patients with degenerative diseases. Although we cannot confirm whether similar patterns would be observed in other trials, our study highlights the importance of assessing whether results from randomized trials can be generalized more broadly, which may benefit the implementation of findings in clinical practice.

A recent study found a mean preoperative OHS of 20 points across international THA registries [20]. This is lower than the mean OHS baseline values in both groups of our current study. On one hand, it could indicate limited representativeness of the patients in the non-PROHIP cohort, suggesting that the patients enrolled in the PROHIP trial had clinically meaningful lesser hip pain and better function at baseline than the general population with severe hip osteoarthritis undergoing THA worldwide. On the other hand, the observed baseline values for the OHS in the patients in the PROHIP trial and non-PROHIP cohort may be explained by national variation, as the mean preoperative OHS ranges from 18 to 23 points across registries [20]. In this regard, the observed baseline values for the OHS for both groups are comparable to those previously reported by patients in Australia, the Netherlands, and Denmark [20,21]. Furthermore, the distribution of sex, age, and body mass index in both groups in the current study was similar to those of patients undergoing THA across the world [20,21]. This supports the generalizability and external validity of our main results from the PROHIP trial [5].

Despite conducting a qualitative patient and public involvement study to improve our trial design, patient information, and recruitment procedures [22], our enrollment rate in PROHIP was only 7%. This is similar to the 7% enrollment rate from a previous randomized controlled trial comparing TKA with first-line treatment in patients with knee OA, which did not use patient and public involvement [8]. This suggests that our strategy had little impact on the enrollment rates, contrasting with previous meta-analysis results [23]. The limited impact may also be due to the COVID-19 pandemic, which made patients less likely to enroll in randomized controlled trials even when hospitals returned to pre-pandemic status [24]. Overall, our low enrollment rate in the PROHIP trial highlights the challenge of recruiting patients for this type of randomized controlled trial comparing surgery with that of first-line treatment, as observed previously [6-8].

Although exercise is consistently recommended as part of the first-line treatment for hip osteoarthritis [4], previous findings indicate that only about 40% of the patients are either recommended or referred to exercise in clinical practice [25]. This is consistent with the proportion of patients in the non-PROHIP cohort who had previously attended supervised exercise, but higher than the proportion of patients enrolled in the PROHIP trial. This supports our findings that indicate reduced representativeness of experience with previous first-line treatment among patients enrolled in the PROHIP trial. This may have affected our estimates of treatment effect, possibly having led to an overestimation of resistance training [5].

### Limitations

A key limitation was the low enrollment rate in the non-PROHIP cohort, where only 293 (43%) out of 681 eligible patients accepted participation and met the inclusion criteria such as to be able to understand Danish. This may have reduced the representativeness of the non-PROHIP cohort and influenced our interpretation of the results, particularly regarding the generalizability of the PROHIP trial [5]. However, the patients in the non-PROHIP cohort had similar demographics and OHS at baseline compared with previous representative cohorts of patients undergoing THA [20,21], which supports the robustness of our findings despite the low enrollment rate. Another limitation is that we obtained patient-reported outcome scores only from patients in the non-PROHIP cohort. As a result, we could not assess potential important differences in the functional performance tests recommended by the Osteoarthritis Research Society International (OARSI) [26]. Nevertheless, the patient-reported outcome measures used are valid, reliable, and responsive, covering the Outcome Measures in Rheumatology–Osteoarthritis Research Society International (OMERACT-OARSI) core domain set for measurement in randomized trials [27], thereby reflecting meaningful measures for patients with hip osteoarthritis, clinicians, and decision-makers. A further limitation involves the slight procedural differences in data collection between the 2 groups. Patients in the PROHIP trial completed electronic questionnaires in a hospital setting with access to clarifications and had height and weight measured by an outcome assessor, while patients in the non-PROHIP cohort completed them at home and self-reported these metrics. These differences may have introduced a minor risk of detection and information bias, potentially affecting our results such as the observed difference in body mass index. However, these procedural variations are unlikely to substantially impact the overall findings, as all other demographics and patient-reported outcome scores were self-reported and consistently collected between both groups.

### Strengths

The strength of our study is that the data is derived from a robust multicenter recruitment effort involving 4 orthopedic departments, which span 3 of the 5 healthcare regions in Denmark. This broad geographic coverage enhances the representativeness and generalizability of our results. Furthermore, we conducted the main analyses in accordance with a predefined statistical analysis plan made publicly available before any analyses commenced. Finally, we used balance diagnostics for comparing the distribution of baseline variables between the 2 groups, as this method is not directly influenced by sample size.

### Conclusion

We found minimal imbalances in most demographic variables and patient-reported outcomes, with those who accepted enrollment in the PROHIP trial reporting less experience with previous joint arthroplasty surgery and supervised exercise and slightly more favorable outcomes, indicating less severe symptoms for almost all domains at baseline than those who declined. However, most differences were probably not clinically important. Although caution is warranted due to the potentially limited representativeness of the non-PROHIP group, these findings support the generalizability of the PROHIP trial. This highlights that the findings from the PROHIP trial appear applicable to broader clinical practice.

### Supplementary data

The study protocol is available as Supplementary data on the article page, doi: 10.2340/17453674.2025.44756 and 10.1136/bmjopen-2021-051392

## Supplementary Material



## Notes

Conception and design: TF, RC, IM, LRM, SO, and KGI. Obtaining of funding: TF, IM, LRM, SO, and KGI. Collection and assembly of data: TF, CV, MJB, and SSJ. Data analysis and interpretation: TF and RC. Writing the first draft of the manuscript: TF. Critical revision of the manuscript for important intellectual content and final approval: TF, RC, IM, LRM, CV, MJB, SSJ, SO, and KGI.

The authors would like to thank all the patients who participated in this study. They also wish to acknowledge the orthopedic surgeons and research coordinators from the Department of Orthopedic Surgery, University Hospital of Southern Denmark, Vejle Hospital; Department of Orthopedic Surgery, Næstved Hospital; Department of Orthopedic Surgery and Traumatology, Odense University Hospital (OUH); and Department of Orthopedic Surgery, Aarhus University Hospital (AUH) for their involvement in the enrollment of patients. Lastly, the authors would like to acknowledge the Odense Patient data Explorative Network (OPEN), Odense University Hospital, Odense, Denmark, for data management support and access to REDCap.

Handling co-editors: Bart A Swierstra and Philippe Wagner

*Acta* thanks David N Bernstein and Babette C van der Zwaard for help with peer review of this manuscript.
