# Supplementary material for: Generalizability of the Progressive Resistance Training versus Total Hip Arthroplasty (PROHIP) trial: a cross-sectional study of 402 patients in Denmark
Source: Acta Orthop. 2025 Sep 17;96:698–705. doi: 10.2340/17453674.2025.44756 (PMC12444794; doi:10.2340/17453674.2025.44756)
Supplement: Supplementary file 1 [file ActaO-96-44756-s1.pdf]

Generalizability of Patients with Severe Hip Osteoarthritis Accepting Participation in a Randomized Trial on Total Hip Arthroplasty and Progressive Resistance Training: *Statistical Analysis Plan for the PROHIP Cross-Sectional Study Comparing Baseline Characteristics and Propensity of Patients Accepting and Declining Enrollment*

Thomas Frydendal[1,2,3], Robin Christensen[3,4] Inger Mechlenburg[5,6], Lone Ramer Mikkelsen[6,7], Søren Overgaard*[8,9,10], Kim Gordon Ingwersen*[1,3,11]

Affiliations:

[1]Department of Physiotherapy, Lillebaelt Hospital – University Hospital of Southern Denmark, Vejle Hospital, Denmark.

[2]Department of Clinical Research, University of Southern Denmark, Odense, Denmark.

[3]Section for Biostatistics and Evidence-Based Research, the Parker Institute, Bispebjerg and Frederiksberg Hospital, Copenhagen, Denmark

[4]Research Unit of Rheumatology, Department of Clinical Research, University of Southern Denmark, Odense University Hospital, Odense Denmark

[5]Department of Orthopaedic Surgery, Aarhus University Hospital, Aarhus, Denmark

[6]Department of Clinical Medicine, Aarhus University, Aarhus, Denmark

[7]Elective Surgery Centre, Silkeborg Regional Hospital, Silkeborg, Denmark

[8]Department of Orthopaedic Surgery and Traumatology, Odense University Hospital, Odense, Denmark

[9]Department of Orthopaedic Surgery and Traumatology, Copenhagen University Hospital, Bispebjerg Hospital, Copenhagen, Denmark

[10]Department of Clinical Medicine, Faculty of Health and Medical Sciences University of Copenhagen, Copenhagen, Denmark

[11]Department of Regional Health Research, University of Southern Denmark, Odense, Denmark.

Principle Investigator: Thomas Frydendal (TF), Department of Physiotherapy, Lillebaelt Hospital – University Hospital of Southern Denmark, Vejle Hospital, Denmark and Department of Clinical Research, University of Southern Denmark, Odense, Denmark.

Senior Biostatistician and Analysis Support: Robin Christensen (RC), Professor of Biostatistics & Clinical Epidemiology: Section for Biostatistics and Evidence-Based Research, the Parker Institute, Bispebjerg and Frederiksberg Hospital, Copenhagen, Denmark and Research Unit of Rheumatology, Department of Clinical Research, University of Southern Denmark, Odense University Hospital, Odense Denmark

Statistical Analyst: Thomas Frydendal (TF), Department of Physiotherapy, Lillebaelt Hospital – University Hospital of Southern Denmark, Vejle Hospital, Denmark and Department of Clinical Research, University of Southern Denmark, Odense, Denmark.

Database Manager: David Hass (DH), Open Patient data Explorative Network (OPEN), Odense University Hospital, Region of Southern Denmark

Trial Registration Number: ClinicalTrials.gov (NCT04070027)

Protocol Version: This document has been written based on the information contained in the PROHIP trial protocol version 1.3, dated 22th of June 2021.

Statistical Analysis Plan (SAP) version: The PROHIP Generalizability SAP version 1.0, dated 3rd of August 2022.

SIGNATURES

Date	Name	Roles and Responsibility	Signature
Aug. 3, 2022	Thomas Frydendal	SAP first author<br>(Team leader)	
Aug. 3, 2022	Robin Christensen	Senior Biostatistician<br>(Risk Manager)	
Aug. 3, 2022	Søren Overgaard	Chief Investigator<br>(Sponsor)	
Aug. 3, 2022	Kim Gordon Ingwersen	SAP last author<br>(Project leader)	

INTRODUCTION

Background and rationale

Hip osteoarthritis (OA) is the leading cause for undergoing total hip arthroplasty (THA)[1]. This procedure is widely acknowledged to be highly effective for reducing hip pain, and improving functional outcomes and quality-of-life in the management of severe hip OA[2-4]. Around 90-95% of the patients are satisfied with the outcome one year postoperatively[5], but one fifth of the patients undergoing THA report residual pain after surgery[6]. This may possibly be due to significant variation in the indication criteria for THA[7-9]. Moreover, patient willingness to undergo surgery has been shown as the strongest predictor for undergoing THA[10], which could indicate a need for a more objective criteria.

Exercise is recommended as first-line treatment in the management of mild to moderate hip OA[11-13]. Supervised progressive resistance training (PRT) has shown a moderate effect for improving multiple outcomes and may be of clinical relevance even in patients with severe hip OA[14, 15]. Furthermore, first-line treatment comprising exercise and patient education may postpone the need for surgery and reduce the willingness to undergo THA[16, 17].

The PROgressive resistance training versus total HIP arthroplasty (PROHIP) trial aims to determine if THA surgery is a superior treatment to PRT in patients suffering from severe hip OA[18]. However, previous clinical trials comparing surgical procedures with first-line treatment have had enrollment rates between 7% and 22%[19-23]. Low enrollment rates may decrease the generalizability of these trials making it unclear to which patients the result can be applied[24-27].

Objectives

The primary objective of this study is to compare baseline characteristics of patients with severe hip OA eligible for THA *accepting versus* those *declining* enrollment in the PROHIP trial in order to evaluate whether enrolled patients differ systematically (i.e. bias) from the patients declining participation in hip pain and function, measured using the Oxford Hip Score (OHS), assessed at baseline.

The primary null hypothesis is that there is no difference in hip pain and function between the two groups. The alternative hypothesis is that patients declining enrollment in the PROHIP trial have significantly worse hip pain and function than those accepting participation.

Secondary objectives are:

1) To compare baseline characteristics of patients with severe hip OA eligible for THA *accepting versus* those *declining* enrollment in the PROHIP trial in order to evaluate whether enrolled patients differ systematically from the patients declining participation in hip pain, hip symptoms, activities-of-daily-living (ADL) function, hip-related quality-of-life (QoL), and sport and recreation function (sport/recreation), measured using the Hip disability and Osteoarthritis Outcome Score (HOOS) subscales, assessed at baseline.
2) To compare baseline characteristics of patients with severe hip OA eligible for THA *accepting versus* those *declining* enrollment in the PROHIP trial in order to evaluate whether enrolled patients differ systematically from the patients declining participation in physical activity level, measured using the University of California Los Angeles (UCLA) Activity Score, assessed at baseline.
3) To compare baseline characteristics of patients with severe hip OA eligible for THA *accepting versus* those *declining* enrollment in the PROHIP trial in order to evaluate whether enrolled patients differ systematically from the patients declining participation in pain intensity in the index hip at rest and during activity, measured using the Visual Analogue Scale (VAS), assessed at baseline.
4) To compare baseline characteristics of patients with severe hip OA eligible for THA *accepting versus* those *declining* enrollment in the PROHIP trial in order to evaluate whether enrolled patients differ systematically from the patients declining participation in health-related quality of life, measured using the EuroQol Group 5-dimension 5 level (EQ-5D-5L) including summary index and VAS, assessed at baseline.
5) To compare baseline characteristics of patients with severe hip OA eligible for THA *accepting versus* those *declining* enrollment in the PROHIP trial in order to evaluate whether enrolled patients differ systematically from the patients declining participation in sex, age, height, weight, body mass index (BMI), educational level, employment status, smoking status, alcohol consumption, hip symptoms duration, previous THA and total knee arthroplasty (TKA), medicine consumption, and number of comorbidities, measured using a patient-reported questionnaire, assessed at baseline.

STUDY METHODS

Study design

The PROHIP trial was designed as a multicenter (four sites), stratified (by site), randomized (allocation 1:1), controlled, parallel-group superiority trial. Patients were recruited from the orthopedic departments at Vejle Hospital and Odense University Hospital (OUH) in the Region of Southern Denmark, Aarhus University Hospital (AUH) in the Central Denmark Region, and Næstved Hospital in Region Zealand and randomized to THA followed by standard care or 12-weeks of supervised PRT followed by 12-weeks of optional unsupervised PRT. The treatments are described in full details in the trial protocol[18]. Patients who declined participation in the PROHIP trial was invited into a parallel prospective cohort. This study was designed as a cross-sectional study using patient-reported baseline characteristics and outcomes of patients with severe hip OA eligible for enrollment in the PROHIP trial who either accepted (PROHIP group) or declined (non-PROHIP group) participation in the clinical trial. Patient enrolment and data collection was started on the September 2nd 2019 and ended on June 30th 2021 as the final deadline for recruitment was reached for the clinical trial[18].

The PROHIP trial protocol was reported in accordance with the *'Standard Protocol Items: Recommendations for Interventional Trials'* (SPIRIT) statement[28]. This statistical analysis plan (SAP) adheres to the *'Guidelines for the Content of Statistical Analysis Plans in Clinical Trials'*[29], while reporting of the cross-sectional study will follow the *'Strengthening the Reporting of Observational Studies in Epidemiology'* (STROBE) statement[30].

Randomization

The randomization process for the PROHIP trial is described in full details in the trial protocol[18]. No randomization was performed in the present study, since this is an observational study comparing patients who accepted with those who declined participation in the PROHIP trial.

Sample size and power calculation

The sample size and power calculation for the PROHIP trial is described in full details in the trial protocol[18]. For the present cross-sectional study, no formal sample size calculation was performed and patients for the observational cohort was enrolled prospectively and consecutively until enrollment for the PROHIP trial ended (i.e. the ratio of patients who accepted versus those who declined is unclear, and will be an important outcome of the present study).

Statistical interim analysis and stopping guidance

No formal statistical interim analysis is planned on the primary endpoint (baseline difference for the OHS score between the two groups [PROHIP and non-PROHIP]). The final deadline for patient recruitment was a priori set 18 months (i.e. February 2021) after the inclusion of patients was started. However, the recruitment deadline was prolonged 4 months to June 2021 due to the COVID-19 lockdown in Denmark in March 2020. The author group have monitored recruitment and attrition rates in the study.

Timing of final analysis

The final analysis for the between-group comparison (PROHIP versus non-PROHIP) for the primary endpoint (baseline) is planned complement of baseline measurements and the final deadline for recruitment is reached for the clinical trial[18]. The publication of this cross-sectional study will be prepared when these data have been retrieved and cleaned (anticipated by August 2022).

Timing of outcome assessments

The overview of PROHIP trial procedures and time-point of each outcome assessment is presented in the Table 3 in the trial protocol[18]. This cross-sectional comparison between PROHIP and non-PROHIP participants evaluates patients-reported outcomes measured at baseline. For the PROHIP group, patient-reported questionnaires had to be completed prior to randomization in the PROHIP trial in order to be included in the analysis. For the non-PROHIP group, patient-reported questionnaires had to be completed prior to THA surgery in order to be included in the analysis.

STATISTICAL PRINCIPLES

Confidence intervals and p-values

All *P* values and confidence intervals will be two-sided; *p-values* <0.05 will be considered statistically significant. For the OHS, a 95% confidence interval excluding a difference greater than 5 OHS points between the two groups will be interpreted as indicating absence of a minimal clinically important difference (i.e. possible equivalence). No adjustment for multiplicity will be performed.

Adherence and protocol deviations

Treatment adherence in both the PROHIP and non-PROHIP group will not be presented due the cross-sectional design with the objective to compare baseline characteristics.

The following are pre-defined major protocol deviations: (1) patients in the PROHIP group not completing the patient-reported questionnaires prior to randomization, (2) patients in the non-PROHIP group not completing the patient-reported questionnaires prior to THA surgery, and (3) patients in both groups withdrawing from the study between inclusion and baseline assessment. The number (percentage) of patients with major protocol deviations will be summarized by group. No formal statistical testing will be conducted.

Analysis populations

The primary analyses will be based on a per-protocol population. The per-protocol analysis set consists of patients from the PROHIP and non-PROHIP groups with a baseline measurement on the patient characteristics questionnaire, primary outcome, key secondary outcomes, and exploratory outcomes.

STUDY POPULATION

Screening data

The following enrolment data will be presented for each hospital for each month during the study period: (1) the number of patients assessed for eligibility, (2) the number of patients enrolled (PROHIP and non-PROHIP), and (3) enrollment rate. This detailed enrollment summary is illustrated in **Appendix Figure 1** and **Appendix Figure 2**.

Eligibility

Patients conforming to the following inclusion and exclusion criteria are considered eligible for the PROHIP trial and the current cross-sectional study.

Inclusion criteria: (1) Patients aged ≥50 years; (2) Clinical history and symptoms consistent with primary hip OA (including hip OA due to mild hip dysplasia that may be treated with standard components) and radiographic verified hip OA defined as joint space narrowing <2 mm; (3) Considered eligible for THA by an orthopedic surgeon (i.e. hip-related pain, symptom duration >3 months, functional impairment or decreased range-of-motion, and attempted treatment with analgesics).

Exclusion criteria: (1) Severe walking deficits (i.e. dependency of two crutches or walker); (2) Body Mass Index (BMI) >35 kg/m^2; (3) Lower extremity fractures within previous 12 months; (4) Planned other lower extremity surgery within 6 months; (5) Cancer diagnosis and current chemo-, immuno- or radiotherapy; (6) Neurological diseases (e.g. previous stroke, multiple

sclerosis, Parkinson's, Alzheimer's); (7) Other reasons for exclusion (i.e. inadequacy in written and spoken Danish, mentally unable to participate, physically unable to comply with the PRT protocol due to comorbidity (e.g. severe heart disease, previous major lower extremity surgery within previous 6 months) etc.).

The total number of patients screened for eligibility from the four hospitals will be collected and presented in a CONSORT flowchart to describe representativeness of the study sample. Furthermore, the number of ineligible patients will be reported including reason for ineligibility.

Recruitment

The CONSORT flowchart will comprise number of patients screened assessed for eligibility, excluded due to ineligibility (with reasons), assessed eligible, declined participation in PROHIP, declined participation in cross-sectional study, accepted inclusion in PROHIP, accepted inclusion in non-PROHIP, withdrawals (with reasons), and included in the per-protocol analysis. The CONSORT flowchart is depicted in **Figure 1.**

Withdrawal/follow-up

The level of consent withdrawal will be classified by the following two options: (1) consent to continue follow-up and data collection and (2) complete withdrawal with no further follow-up and data collection.

Timing of withdrawal and loss to follow-up will be presented in the CONSORT flowchart with numbers and reasons for withdrawal and/or loss to follow-up given at the baseline (primary end point) outcome assessment. Furthermore, the number (with reasons) of loss to follow-up during the course of the study will summarized by group.

Baseline patient characteristics

The following data will be used to describe patients by group (PROHIP and non-PROHIP) at baseline: sex, age, height, weight, BMI, educational level beyond high school, employment status, alcohol consumption above Danish recommendations[31], index hip, duration of hip symptoms, previous THA, previous total knee arthroplasty, use of analgesics in the previous week, comorbidities, OHS, HOOS subscales (pain, symptoms, ADL function, sport/rec, hip-related QoL), UCLA activity score, VAS pain (rest), VAS pain (activity), EQ-5D-5L index score, and EQ-VAS score.

Numbers and percentages will be calculated and presented for categorical variables. Means and standard deviations (SD) will be computed and presented for continues variables if data follows a normal distribution. The baseline characteristics will be presented as illustrated in **Table 1.**

ANALYSIS

Outcome definitions

Primary outcome

Oxford Hip Score (OHS)

The primary outcome measure will be the between-group difference at baseline ($OHS_{baseline}$). The OHS is considered a valid, reliable, and responsive patient-reported questionnaire assessing hip pain and function in a composite score ranging from 0 (worst) to 48 (best)[32 33-36].

Key secondary outcomes

Hip disability and Osteoarthritis Outcome Score (HOOS)

A key secondary outcome will be the between-group difference at baseline in each HOOS subscale ($HOOS_{baseline}$). The HOOS is a valid, reliable and responsive patient-reported questionnaire consisting of five subscales covering, hip pain, hip symptoms, ADL function, hip-related QoL, and sport/recreation with each subscale score ranging from 0 (worst) to 100 (best)[37-40].

University of California Los Angeles (UCLA) Activity Score

A key secondary outcome will be the between-group difference at baseline in UCLA activity score (UCLA activity $score_{baseline}$). The UCLA is reliable, valid, and responsive measure of patient-reported physical activity level ranging from 1 (inactive) to 10 (regular participation in impact sport or heavy labour)[41-43].

Exploratory outcomes

Visual Analogue Scale (VAS)

An exploratory outcome will be the between-group difference at baseline in VAS hip pain intensity (VAS $rest_{baseline}$ and VAS $activity_{baseline}$). The VAS is a reliable, valid and responsive measure of patient-reported pain intensity ranging from 0 (no pain) to 100 (worst pain imaginable)[44].

EuroQol Group 5-dimension (EQ-5D-5L)

An exploratory outcome will be the between-group difference at baseline in EQ-5D-5L (EQ-5D-5L $index_{baseline}$ and $EQ\text{-}VAS_{baseline}$). The EQ-5D-5L is a reliable and valid measure of patient-reported health-related quality-of-life including the summary index ranging from -0.624 (worst) to 1.000 (best) (Danish value set) and EQ-VAS ranging from 0 (worst imaginable health) to 100 (best imaginable health)[45-49]. The major outcomes will be presented as illustrated in **Table 1.**

Analysis methods

All descriptive statistics and statistical analysis will be reported in accordance with the recommendations of the *'Enhancing the QUAlity and Transparency Of health Research'* (EQUATOR) network[50] and the CONSORT statement.[51] Visual inspection (QQ-plot, histograms, and scatterplots) will be used to assess the assumption of normality of continuous variables.

The term balance diagnostics will be used to describe the statistical methods applied to evaluate whether the distribution of baseline covariates is similar between the PROHIP and non-PROHIP groups. Means and/or medians of continuous variables and the distribution of categorical variables will be reported for each of the two groups. These crude comparisons between PROHIP and non-PROHIP allow an evaluation of the comparability of the two groups, which will provide an indication of the generalizability of the PROHIP trial[52].

Descriptive Statistical Measures: Between-group comparisons of continuous variables will be estimated using standardized differences defined as:

$$d = \frac{\bar{x}_{PROHIP} - \bar{x}_{non-PROHIP}}{\sqrt{\frac{s^2_{PROHIP} + s^2_{non-PROHIP}}{2}}}$$

Where $\bar{X}$ denotes the sample means of the variable in PROHIP and non-PROHIP participants, respectively, whereas the s^2 represents the sample variance of the variables in the groups. Between-group comparisons of categorical variables will be estimated using standardized differences defined as:

$$d = \frac{\hat{p}_{PROHIP} - \hat{p}_{non-PROHIP}}{\sqrt{\frac{\hat{p}_{PROHIP}(1 - \hat{p}_{non-PROHIP}) + \hat{p}_{PROHIP}(1 - \hat{p}_{non-PROHIP})}{2}}}$$

Where $\hat{p}$ denotes the prevalence or mean of the categorical variable in PROHIP and non-PROHIP participants, respectively. A standardized difference of ≥0.2 will be used to indicate that there might

be a difference in the baseline variable (i.e. potentially low generalizability), while a standardized difference of ≥0.8 will be considered as a definitive difference.

Inferential Statistical Measures: The SAS PROC $NPAR_1WAY$ procedure will be used to compute the empirical function (EDF) statistics to test the distribution of baseline variables. This procedure provides a summary of the Wilcoxon scores for the analysis of the baseline variable by group level (PROHIP and non-PROHIP) and displays the one-way ANOVA statistics. If the *P-value* is <0.05 this indicates that there might be a difference between the groups (i.e. leads to rejection of the null hypothesis that there are no difference between the groups).

Logistic regression will be used to develop propensity scores, which represent the probability that a patient accept participation in the PROHIP trial depending on the patients observed covariates. Group status (PROHIP and non-PROHIP) will be the dependent variable and baseline variables the covariates.

Missing data

No imputation will be conducted as patients with missing data on the patient characteristics questionnaire, primary outcome, key secondary outcomes, and exploratory outcomes will be considered ineligible for the analysis of the missing individual variables.

Additional analyses

No additional analyses on the primary outcome and key secondary and exploratory outcomes are planned at baseline.

Harms

No summary of adverse events (AEs) will be performed for this study due to the cross-sectional design using data obtained from patient-reported questionnaires measured at baseline.

Statistical software

All statistical analyses and calculations will be performed using SAS (SAS Institute Inc., Cary, North Carolina, USA) and/or STATA (Statacorp, College Station, Texas, USA).

References

1. Ferguson RJ, Palmer AJ, Taylor A, Porter ML, Malchau H, Glyn-Jones S. Hip replacement. Lancet 2018; 392: 1662-1671.

2. Learmonth ID, Young C, Rorabeck C. The operation of the century: total hip replacement. Lancet 2007; 370: 1508-1519.

3. Rosenlund S, Broeng L, Holsgaard-Larsen A, Jensen C, Overgaard S. Patient-reported outcome after total hip arthroplasty: comparison between lateral and posterior approach. Acta Orthop 2017; 88: 239-247.

4. Shan L, Shan B, Graham D, Saxena A. Total hip replacement: a systematic review and meta-analysis on mid-term quality of life. Osteoarthritis Cartilage 2014; 22: 389-406.

5. Paulsen A, Roos EM, Pedersen AB, Overgaard S. Minimal clinically important improvement (MCII) and patient-acceptable symptom state (PASS) in total hip arthroplasty (THA) patients 1 year postoperatively. Acta Orthop 2014; 85: 39-48.

6. Beswick AD, Wylde V, Gooberman-Hill R, Blom A, Dieppe P. What proportion of patients report long-term pain after total hip or knee replacement for osteoarthritis? A systematic review of prospective studies in unselected patients. BMJ Open 2012; 2: e000435.

7. Ackerman IN, Dieppe PA, March LM, Roos EM, Nilsdotter AK, Brown GC, et al. Variation in age and physical status prior to total knee and hip replacement surgery: a comparison of centers in Australia and Europe. Arthritis Rheum 2009; 61: 166-173.

8. Cobos R, Latorre A, Aizpuru F, Guenaga JI, Sarasqueta C, Escobar A, et al. Variability of indication criteria in knee and hip replacement: an observational study. BMC Musculoskelet Disord 2010; 11: 249.

9. Dreinhöfer KE, Dieppe P, Stürmer T, Gröber-Grätz D, Flören M, Günther KP, et al. Indications for total hip replacement: comparison of assessments of orthopaedic surgeons and referring physicians. Ann Rheum Dis 2006; 65: 1346-1350.

10. Hawker GA, Guan J, Croxford R, Coyte PC, Glazier RH, Harvey BJ, et al. A prospective population-based study of the predictors of undergoing total joint arthroplasty. Arthritis Rheum 2006; 54: 3212-3220.

11. Brosseau L, Wells GA, Pugh AG, Smith CA, Rahman P, Àlvarez Gallardo IC, et al. Ottawa Panel evidence-based clinical practice guidelines for therapeutic exercise in the management of hip osteoarthritis. Clin Rehabil 2016; 30: 935-946.

12. Danish Health Authority. National Clinical Guideline on Hip Osteoarthritis - Non-surgical Treatment and Rehabilitation Following Total Hip Arthroplasty. 2nd ed: Danish Health Authority 2021.

13. van Doormaal MCM, Meerhoff GA, Vliet Vlieland TPM, Peter WF. A clinical practice guideline for physical therapy in patients with hip or knee osteoarthritis. Musculoskeletal Care 2020.

14. Goh SL, Persson MSM, Stocks J, Hou Y, Welton NJ, Lin J, et al. Relative Efficacy of Different Exercises for Pain, Function, Performance and Quality of Life in Knee and Hip Osteoarthritis: Systematic Review and Network Meta-Analysis. Sports Med 2019; 49: 743-761.

15. Hansen S, Mikkelsen LR, Overgaard S, Mechlenburg I. Effectiveness of supervised resistance training for patients with hip osteoarthritis - a systematic review. Dan Med J 2020; 67.

16. Dell'Isola A, Jönsson T, Rolfson O, Cronström A, Englund M, Dahlberg L. Willingness to Undergo Joint Surgery Following a First-Line Intervention for Osteoarthritis: Data From the Better Management of People With Osteoarthritis Register. Arthritis Care Res (Hoboken) 2021; 73: 818-827.

17. Svege I, Nordsletten L, Fernandes L, Risberg MA. Exercise therapy may postpone total hip replacement surgery in patients with hip osteoarthritis: a long-term follow-up of a randomised trial. Ann Rheum Dis 2015; 74: 164-169.

18. Frydendal T, Christensen R, Mechlenburg I, Mikkelsen LR, Overgaard S, Ingwersen KG. Total hip arthroplasty versus progressive resistance training in patients with severe hip osteoarthritis: protocol for a multicentre, parallel-group, randomised controlled superiority trial. BMJ Open 2021; 11: e051392.

19. Frobell RB, Roos EM, Roos HP, Ranstam J, Lohmander LS. A randomized trial of treatment for acute anterior cruciate ligament tears. N Engl J Med 2010; 363: 331-342.

20. Skou ST, Roos EM, Laursen MB, Rathleff MS, Arendt-Nielsen L, Simonsen O, et al. A Randomized, Controlled Trial of Total Knee Replacement. N Engl J Med 2015; 373: 1597-1606.

21. van de Graaf VA, Noorduyn JCA, Willigenburg NW, Butter IK, de Gast A, Mol BW, et al. Effect of Early Surgery vs Physical Therapy on Knee Function Among Patients With

Nonobstructive Meniscal Tears: The ESCAPE Randomized Clinical Trial. Jama 2018; 320: 1328-1337.

22. Weinstein JN, Tosteson TD, Lurie JD, Tosteson AN, Hanscom B, Skinner JS, et al. Surgical vs nonoperative treatment for lumbar disk herniation: the Spine Patient Outcomes Research Trial (SPORT): a randomized trial. Jama 2006; 296: 2441-2450.

23. Skou ST, Hölmich P, Lind M, Jensen HP, Jensen C, Garval M, et al. Early Surgery or Exercise and Education for Meniscal Tears in Young Adults. NEJM Evidence 2022; 1.

24. Canevelli M, Trebbastoni A, Quarata F, D'Antonio F, Cesari M, de Lena C, et al. External Validity of Randomized Controlled Trials on Alzheimer's Disease: The Biases of Frailty and Biological Aging. Front Neurol 2017; 8: 628.

25. Collins GS, de Groot JA, Dutton S, Omar O, Shanyinde M, Tajar A, et al. External validation of multivariable prediction models: a systematic review of methodological conduct and reporting. BMC Med Res Methodol 2014; 14: 40.

26. Kennedy-Martin T, Curtis S, Faries D, Robinson S, Johnston J. A literature review on the representativeness of randomized controlled trial samples and implications for the external validity of trial results. Trials 2015; 16: 495.

27. van Deudekom FJ, Postmus I, van der Ham DJ, Pothof AB, Broekhuizen K, Blauw GJ, et al. External validity of randomized controlled trials in older adults, a systematic review. PLoS One 2017; 12: e0174053.

28. Chan AW, Tetzlaff JM, Gotzsche PC, Altman DG, Mann H, Berlin JA, et al. SPIRIT 2013 explanation and elaboration: guidance for protocols of clinical trials. Bmj 2013; 346: e7586.

29. Gamble C, Krishan A, Stocken D, Lewis S, Juszczak E, Doré C, et al. Guidelines for the Content of Statistical Analysis Plans in Clinical Trials. Jama 2017; 318: 2337-2343.

30. Vandenbroucke JP, von Elm E, Altman DG, Gotzsche PC, Mulrow CD, Pocock SJ, et al. Strengthening the Reporting of Observational Studies in Epidemiology (STROBE): explanation and elaboration. Ann Intern Med 2007; 147: W163-194.

31. Danish Health Authority. Sundhedsstyrelsens udmeldinger om indtag af alkohol. Danish Health Authority 2022.

32. Dawson J, Fitzpatrick R, Carr A, Murray D. Questionnaire on the perceptions of patients about total hip replacement. J Bone Joint Surg Br 1996; 78: 185-190.

33. Murray DW, Fitzpatrick R, Rogers K, Pandit H, Beard DJ, Carr AJ, et al. The use of the Oxford hip and knee scores. J Bone Joint Surg Br 2007; 89: 1010-1014.

34. Beard DJ, Harris K, Dawson J, Doll H, Murray DW, Carr AJ, et al. Meaningful changes for the Oxford hip and knee scores after joint replacement surgery. J Clin Epidemiol 2015; 68: 73-79.

35. Harris K, Dawson J, Gibbons E, Lim CR, Beard DJ, Fitzpatrick R, et al. Systematic review of measurement properties of patient-reported outcome measures used in patients undergoing hip and knee arthroplasty. Patient Relat Outcome Meas 2016; 7: 101-108.

36. Paulsen A, Odgaard A, Overgaard S. Translation, cross-cultural adaptation and validation of the Danish version of the Oxford hip score: Assessed against generic and disease-specific questionnaires. Bone Joint Res 2012; 1: 225-233.

37. de Groot IB, Reijman M, Terwee CB, Bierma-Zeinstra SM, Favejee M, Roos EM, et al. Validation of the Dutch version of the Hip disability and Osteoarthritis Outcome Score. Osteoarthritis Cartilage 2007; 15: 104-109.

38. Klassbo M, Larsson E, Mannevik E. Hip disability and osteoarthritis outcome score. An extension of the Western Ontario and McMaster Universities Osteoarthritis Index. Scand J Rheumatol 2003; 32: 46-51.

39. Nilsdotter AK, Lohmander LS, Klassbo M, Roos EM. Hip disability and osteoarthritis outcome score (HOOS)--validity and responsiveness in total hip replacement. BMC Musculoskelet Disord 2003; 4: 10.

40. Thorborg K, Roos EM, Bartels EM, Petersen J, Holmich P. Validity, reliability and responsiveness of patient-reported outcome questionnaires when assessing hip and groin disability: a systematic review. Br J Sports Med 2010; 44: 1186-1196.

41. Amstutz HC, Thomas BJ, Jinnah R, Kim W, Grogan T, Yale C. Treatment of primary osteoarthritis of the hip. A comparison of total joint and surface replacement arthroplasty. J Bone Joint Surg Am 1984; 66: 228-241.

42. Naal FD, Impellizzeri FM, Leunig M. Which is the best activity rating scale for patients undergoing total joint arthroplasty? Clin Orthop Relat Res 2009; 467: 958-965.

43. Terwee CB, Bouwmeester W, van Elsland SL, de Vet HC, Dekker J. Instruments to assess physical activity in patients with osteoarthritis of the hip or knee: a systematic review of measurement properties. Osteoarthritis Cartilage 2011; 19: 620-633.

44. Hawker GA, Mian S, Kendzerska T, French M. Measures of adult pain: Visual Analog Scale for Pain (VAS Pain), Numeric Rating Scale for Pain (NRS Pain), McGill Pain Questionnaire (MPQ), Short-Form McGill Pain Questionnaire (SF-MPQ), Chronic Pain Grade Scale (CPGS), Short Form-36 Bodily Pain Scale (SF-36 BPS), and Measure of Intermittent and Constant Osteoarthritis Pain (ICOAP). Arthritis Care Res (Hoboken) 2011; 63 Suppl 11: S240-252.

45. Herdman M, Gudex C, Lloyd A, Janssen M, Kind P, Parkin D, et al. Development and preliminary testing of the new five-level version of EQ-5D (EQ-5D-5L). Qual Life Res 2011; 20: 1727-1736.

46. Wittrup-Jensen KU, Lauridsen J, Gudex C, Pedersen KM. Generation of a Danish TTO value set for EQ-5D health states. Scand J Public Health 2009; 37: 459-466.

47. Buchholz I, Janssen MF, Kohlmann T, Feng YS. A Systematic Review of Studies Comparing the Measurement Properties of the Three-Level and Five-Level Versions of the EQ-5D. Pharmacoeconomics 2018; 36: 645-661.

48. Conner-Spady BL, Marshall DA, Bohm E, Dunbar MJ, Loucks L, Al Khudairy A, et al. Reliability and validity of the EQ-5D-5L compared to the EQ-5D-3L in patients with osteoarthritis referred for hip and knee replacement. Qual Life Res 2015; 24: 1775-1784.

49. Janssen MF, Pickard AS, Golicki D, Gudex C, Niewada M, Scalone L, et al. Measurement properties of the EQ-5D-5L compared to the EQ-5D-3L across eight patient groups: a multi-country study. Qual Life Res 2013; 22: 1717-1727.

50. Christensen R, Bliddal H, Henriksen M. Enhancing the reporting and transparency of rheumatology research: a guide to reporting guidelines. Arthritis Res Ther 2013; 15: 109.

51. Moher D, Hopewell S, Schulz KF, Montori V, Gotzsche PC, Devereaux PJ, et al. CONSORT 2010 explanation and elaboration: updated guidelines for reporting parallel group randomised trials. Int J Surg 2012; 10: 28-55.

52. Austin PC. Balance diagnostics for comparing the distribution of baseline covariates between treatment groups in propensity-score matched samples. Stat Med 2009; 28: 3083-3107.

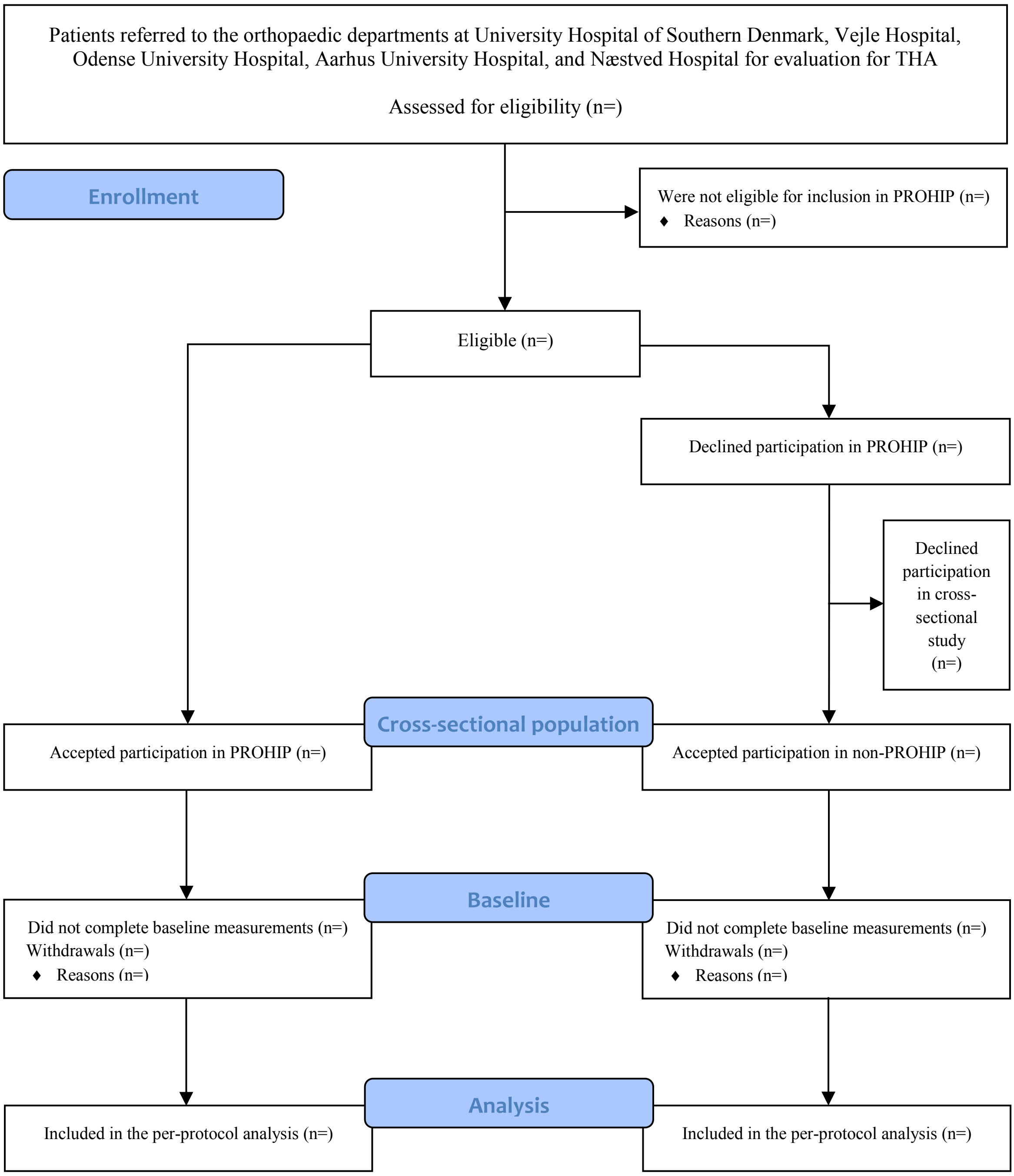

Figure 1. Flowchart of this cross-sectional study. Total hip arthroplasty (THA). The PROgressive resistance training versus total HIP arthroplasty (PROHIP) trial.

Table 1. Baseline characteristics of the patients accepting and declining participation in the PROgressive resistance training versus total HIP arthroplasty (PROHIP) trial.*

	PROHIP (N=??)	Non-PROHIP (N=??)	Between-Group Difference (95% CI)	Standardized Difference
Female sex — no. (%)				
Age — yr				
Height — m				
Weight — kg				
Body-mass index — kg/m^2				
Education level beyond high school — no. (%)				
Employment — no. (%)				
Employed for wages				
Self-employed				
Sick leave				
Retired				
Other				
Substance use — no. (%)				
Current smoker				
Alcohol consumption above recommendations†				
Index hip right — no. (%)				
Duration of hip symptoms — yr				
Previous total hip arthroplasty — no. (%)				
Previous total knee arthroplasty — no. (%)				
Use of analgesics due to hip-related pain — no. (%)				
Paracetamol				
Ibuprofen				
Morphine or opioids				
Other				
Comorbidities — no. (%)				
None				
1				
2				
3 or more				
OHS score‡ — 0 to 48				
HOOS subscale scores§ — 0 to 100				
Pain				
Symptoms				
Function in activities of daily living				
Hip-related quality of life				
Function in sports and recreation				

UCLA activity score¶ — 1 to 10
VAS hip pain at rest‖ — 0 to 100
VAS hip pain during activity‖ — 0 to 100
EQ-5D-5L index score∫ — -0.624 to 1.000
EQ-VAS⁑ — 0 to 100

* Plus–minus values are mean ±SD unless otherwise indicated.

† The Danish Health Authority recommends an alcohol consumption to be no higher than 10 units per week for adults aged 18 or above to have a low risk of developing diseases[31].

‡ The Oxford Hip Score (OHS) ranges from 0 to 48, with higher scores indicating better disease status.

§ For all five subscales, the Hip disability and Osteoarthritis Outcome Score (HOOS) ranges from 0 to 100, with higher scores indicating better disease status.

¶ The University of California Los Angeles (UCLA) Activity Score ranges from 1 to 10, with higher scores indicating greater physical activity level.

‖ The Visual Analogue Scale (VAS) ranges from 0 to 100, with higher scores indicating worse pain intensity.

∫ The EuroQol Group 5-dimension (EQ-5D-5L) index score ranges from -0.624 to 1.000, with higher scores indicating better health-related quality of life.

⁑ The EuroQol Group 5-dimension VAS (EQ-VAS) index score ranges from 0 to 100, with higher scores indicating better health status.

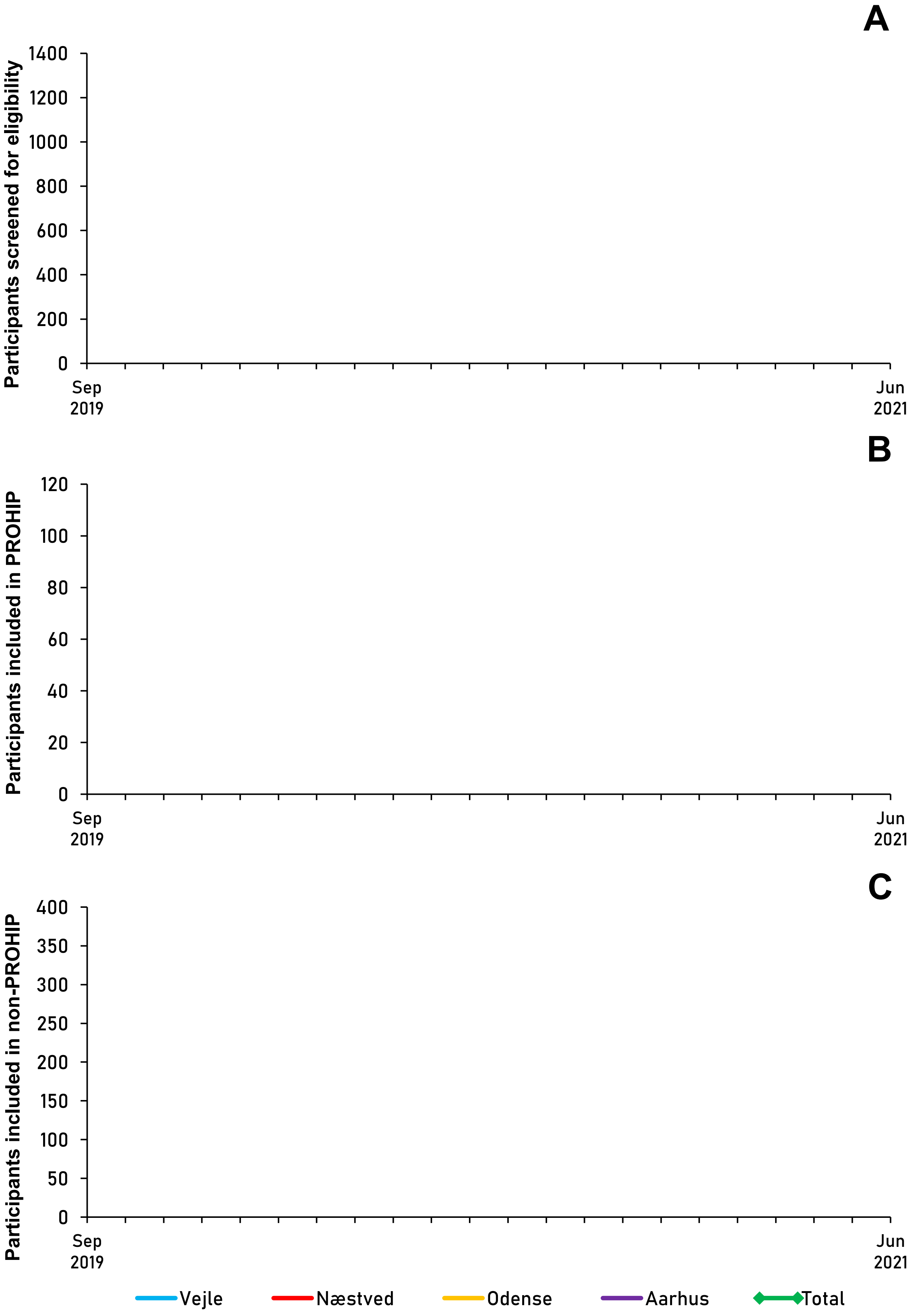

Appendix Figure 1. The number of patients assessed for eligibility (A) and the number of participants enrolled in PROHIP (B) and non-PROHIP (C). The PROgressive resistance training versus total HIP arthroplasty (PROHIP) trial.

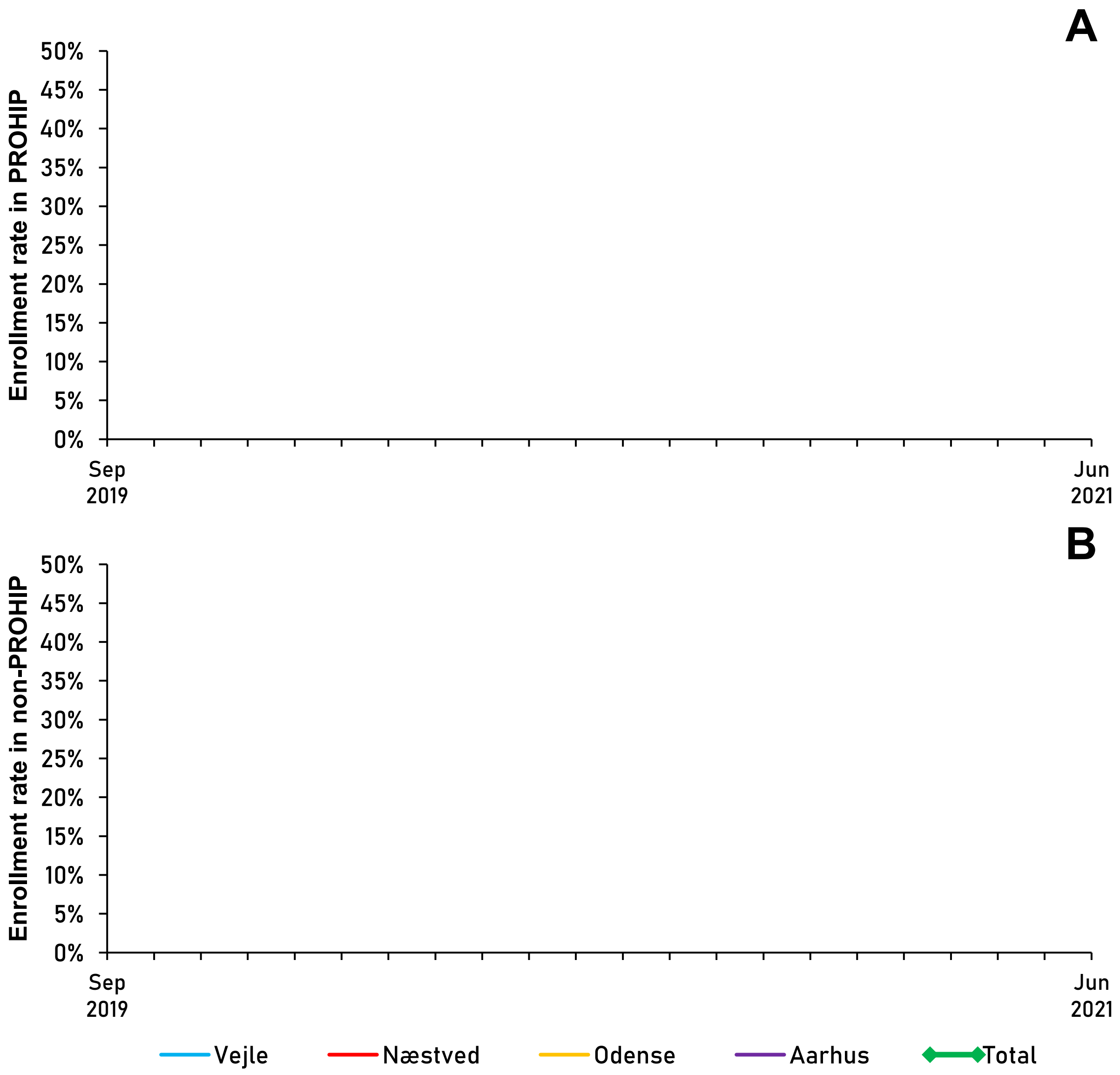

Appendix Figure 2. Enrollment rate in PROHIP (A) and non-PROHIP (B). The PROgressive resistance training versus total HIP arthroplasty (PROHIP) trial.
